# UAV-UGV Collaborative Localisation with Minimum Sensing

**DOI:** 10.3390/s24144629

**Published:** 2024-07-17

**Authors:** A. H. T. Eranga De Silva, Jayantha Katupitiya

**Affiliations:** School of Mechanical and Manufacturing Engineering, University of New South Wales, Sydney, NSW 2052, Australia; j.katupitiya@unsw.edu.au

**Keywords:** constrained Kalman Filter, posterior Cramér–Rao bound, UAV–UGV collaboration, GNSS denied localisation

## Abstract

This paper presents a novel methodology to localise Unmanned Ground Vehicles (UGVs) using Unmanned Aerial Vehicles (UAVs). The UGVs are assumed to be operating in a Global Navigation Satellite System (GNSS)-denied environment. The localisation of the ground vehicles is achieved using UAVs that have full access to the GNSS. The UAVs use range sensors to localise the UGV. One of the major requirements is to use the minimum number of UAVs, which is two UAVs in this paper. Using only two UAVs leads to a significant complication that results an estimation unobservability under certain circumstances. As a solution to the unobservability problem, the main contribution of this paper is to present a methodology to treat the unobservability problem. A Constrained Extended Kalman Filter (CEKF)-based solution, which uses novel kinematics and heuristics-based constraints, is presented. The proposed methodology has been assessed based on the stochastic observability using the Posterior Cramér–Rao Bound (PCRB), and the results demonstrate the successful operation of the proposed localisation method.

## 1. Introduction

Quite often, ground vehicles operate in GNSS-denied environments. In such cases, methodologies are required to enable the localisation of ground vehicles. This paper presents a method to accurately localise the ground vehicles using UAVs. It is assumed that sufficiently accurate GNSS locations of the UAVs are available, and that the UAVs always operate above the tree canopies; for example, localising a UGV that travels under a forest tree canopy can be localised using some UAVs which are flying above the forest tree canopy. In such situations, UAVs can be deployed to collaboratively estimate the location of the UGVs in real time.

The motivations behind developing the research outputs presented in this paper are (1) to develop a UGV localisation method that uses a minimum number of UAVs for UGV localisation; (2) to avoid unobservabilities, which may arise when UAVs are used for UGV localisation; (3) to use the proposed UAV–UGV collaborative system in adverse environment/field conditions. Such UAV–UGV collaborative systems can be used to localise UGVs in battlefields and disaster zones. For example, a UGV can be sent to a high-priority rescue mission where the GNSS reception is weak, and the environment has thick smoke and flames. Moreover, in a rescue scenario during bushfires, such a system can be used to rescue people who are surrounded by bushfires. In such an unfortunate situation, the firetrucks must be operating autonomously, since the firefighters cannot be sent to rescue and the field conditions are adverse. Similarly, UGVs that are used for farming may need the assistance of UAVs for their localisation when their GNSS reception is poor. For example, if a farm is covered by a tall tree canopy, the farming UGVs operating under such a tall tree canopy will not have sufficient GNSS reception. Thereupon, UAVs with sufficient GNSS reception, which hover/fly over the tree canopy, can be used to collaboratively localise farming UGVs operating under the tall tree canopy. Henceforth, a robust localisation method, which has been presented in this paper, is a necessity to navigate such UGVs.

Since there is a motivation to use the proposed UAV–UGV collaborative system in adverse environment/field conditions, sensing methods must be robust against dust, smoke, darkness, high heat, glare, etc. RADAR, LIDAR, Vision Camera, IR camera, and ultrasonic ranging are prospective remote sensing methods. The vision cameras are unable to perform the localisation properly in smoke and also during the night time. Due to the high heat fluxes present, IR cameras will jam the localisation if the localisation is supposed to be performed on a hostile battlefront with frequent glares. Ultrasonic ranging can have accuracy problems due to the impracticality of distinguishing the UGVs clearly from the other sound disturbances. LIDAR seems to show a promising ranging solution, even in vegetation clutter [1], but the inability to penetrate through the dense smoke makes it unsuitable for sensing during a situation like a forest fire [2].

However, RADAR technology shows promising results, as it is not affected by adverse environmental conditions such as bad weather [3] or smoke [4]. RADAR image processing is very cumbersome, and requires expert human intervention to interpret the RADAR scan images. Ultra Wide Band (UWB) sensing is also a RADAR range-finding technique. Unlike RADAR, it does not have to choose a location where surroundings induce minimal clutter, since UWB signals can sense through clutter [5]. In UWB sensing, the large bandwidth enhances reliability as the sensing signal contains different frequencies, which increases the possibility that at least a handful of the emitted signal can go through/around obstacles, and the high bandwidth offers improved ranging accuracy [6]. Moreover, UWB sensors are capable of delivering range measurements at Non-Line-of-Sight (NLOS) situations (e.g., ranging through a forest canopy) without significant degradation of the range measurements [5,7]. Often, UWB sensors can be operated in RADAR mode or range sensing mode. Again, if the UWB sensors are used in the RADAR mode, sensing data processing is very complex, e.g., identifying a UGV travelling under a tree canopy using RADAR mode UWB images, which are acquired from a UAV that is flying above the tree canopy. On the contrary, when UWB sensors are used in the range sensing mode, such data processing complexities do not arise, while the sensing robustness is also safeguarded. Therefore, the proposed UAV–UGV collaborative localisation method is a UWB range-only localisation method.

In UWB ranging, Time of Arrival (TOA) techniques are providing less complex, reliable and cost-effective solutions. There are three commonly used TOA techniques, namely: (i) basic two-way ranging TOA, (ii) synchronous two-way TOA, and (iii) asynchronous two-way TOA. The basic two-way TOA expects ideal instrumentation, which results in low accuracy [6]. In the synchronous two-way TOA method, the time delay in returning back the response to the initial signal sender has been compensated [8]. In addition to the advantages of the synchronous two-way TOA method, the asynchronous two-way TOA method has compensated for frequency and/or phase mismatches between the UWB transceivers [9]. Concerning the aforementioned advantages, UWB range sensing is assumed to be performed by an asynchronous two-way TOA ranging algorithm. Most modern UWB sensors that use the asynchronous two-way ranging method have achieved ranging accuracy up to ±2 cm [7,10]. Henceforth, the observer model in the localisation algorithm does not have to account for either the time delay in range sensing or the frequency and/or the phase mismatches between the UWB transceivers [9].

In a practical application of collaboratively localising a UGV using UAVs, reducing the number of UAVs that have to be utilised for the task is equally important. By reducing the number of UAVs, the capital cost that has to be spent can be reduced. Furthermore, the operational costs can also be reduced since the electricity power cost is low when a lesser number of UAVs are to be airborne. Due to the limited flight time of UAVs, additional UAVs are kept by users to run UAV operations without interruption. The additional UAVs are utilised for the operation while the battery swapping is performed. In that regard, if the number of UAVs required for a UAV operation is minimised, the additional number of UAVs that have to be purchased for the application can also be reduced.

However, as the number of drones is reduced to a minimum of two, processing of the range data for UGV localisation faces substantial challenges. The main problem to be addressed is the ambiguity of the localisation due to the loss of system observability. This paper addresses this problem, and shows the successful localisation of ground vehicles using the proposed method. In the literature, the aforementioned problem is known as the “flip ambiguity phenomenon”, and it has been researched in Wireless Sensor Networks (WSN) and in tracking/localisation. In [11], flip ambiguity has been overcome by using a high number of location anchors/nodes in the WSN so that the ambiguously localised nodes can be identified, and their localisation is supposed to be refined to avoid the flip ambiguity. Since two UAVs are used as anchors in this research, identification of the ambiguous localisation using many UAVs is not possible. In [12], flip ambiguity in intra-localisation of UAVs in a UAV swarm has been addressed, along with the measurement errors. The solution is based on geometric constraints in a 2D plane like in a WSN, which are based on the range measurement constraints, communication range constraints and kinematic information constraints. In [13], an Extended Kalman Filter (EKF) has been designed to localise a GNSS unavailable UAV in a UAV swarm, and the flip ambiguity in localisation has been overcome by estimating the angular velocity of the UAV. However, UAVs have low process noise in their motion. Nevertheless, in a noisy process situation, such as in a UGV motion on a farm/forest ground, angular velocity estimations will have significant deviations, so that granting the angular velocity estimation as crisp information to address the flip ambiguity in localisation will not be a reliable solution to a UGV localisation. Therefore, in this research, a constrained state estimation-based method is developed to address the localisation issues arising from the flip ambiguity.

The system observability was analysed in a deterministic approach to identify the unobservable situations in the proposed localisation method. Based on that, a methodology was developed to successfully avoid the localisation errors caused by the unobservability. In this research, constrained stochastic estimation has been used for localisation. The constraints mitigate the challenges that arise when only two UAVs are utilised for localisation. In order to check the ambiguity aversion performance while using the constrained stochastic location estimation, stochastic observability has been analysed during temporary unobservable scenarios using simulations and experiments.

Due to the strictly/narrowly focused operational scenario considered in this research, the authors did not find comparable past research works/methods that possess similar system implementations. Therefore, the authors believe that the system design of this UAV–UGV collaborative localisation is novel. In the constrained state estimation-based localisation method, all of the kinematics-based constraints are newly formulated for localisation ambiguity aversion. Moreover, when the UGV localisation is performed by UAVs in real time, a method has to be developed to validate the efficacy of the CEKF-based localisation method presented. Thereupon, a novel analytical method is formulated to show the efficacy of the CEKF-based localisation method using the Constrained Posterior Cramér–Rao Bound (CPCRB).

The rest of this paper is organised as follows: Section 2 describes the preliminaries of the motion model, observation model and the EKF-based localisation. Section 3 describes the unobservability identification method of the EKF-based localisation. Section 4 and Section 5 explain the proposed method of overcoming localisation unobservabilities and how the presented unobservability aversion techniques assure the unobservability aversion, respectively. Finally, Section 6 and Section 7 present simulation results and experiment results, respectively.

## 2. Problem Statement

**Figure 1:**
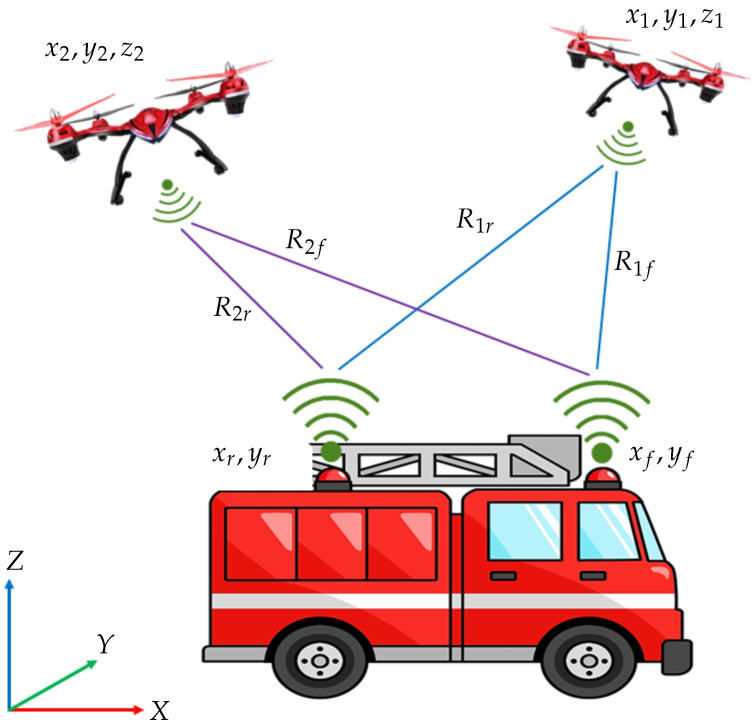
2D localisation of a UGV using drones.

Localisation of a UGV that is travelling on a horizontal planar terrain is supposed to be carried out using two multi-rotor UAVs using range measurements. The overall vehicle and sensor arrangement is as depicted in Figure 1. Since the heading of the UGV is also an important aspect in real-time navigation, two UWB sensors are attached to the ground vehicle at two different locations along the centreline of the ground vehicle at a constant height from the ground. In this paper, the UWB sensors that are attached to the UGV will be named as UWB tags, and are located at $\left(x_{f},y_{f}\right)$ and $\left(x_{r},y_{r}\right)$. Furthermore, UWB range sensors are mounted on each of the drones to obtain the range measurements to two range sensors mounted on the back and on the front of the UGV. UWB sensors that are mounted on each UAV will be named as UWB anchors, and are located at $\left(x_{1},y_{1},z_{1}\right)$ and $\left(x_{2},y_{2},z_{2}\right)$. The UWB anchors that are fixed in the drones fetch the range readings $R_{1f},\;R_{1r},\;R_{2f}$ and $R_{2r}$ between both of the UWB tags on the UGV. For this UGV localisation task, the global positions of the drones are to be known with sufficiently high accuracy. In the next section, the system models that are associated with the localisation are presented.

### 2.1. System Models

#### 2.1.1. Motion Model for UGV Motion Simulation

Taking $x_{a}={\left[x_{a}\;y_{a}\;\theta_{a}\right]}^{T}$ as the state of the UGV in the real world (i.e., simulated actual state), where $x_{a}$ and $y_{a}$ are the geometric centroid’s coordinates of the UGV and $\theta_{a}$ is the heading angle of the UGV in radians, the reduced model of the UGV, by assuming the steering angle ($v_{2}$), speed ($v_{1}$), step time (*T*), and vehicle length (*l*) are known, can be formulated as [14]:

(1) $${\left[\begin{array}{c} x_{a}\\ y_{a}\\ \theta_{a} \end{array}\right]}_{k+1}=\left[\begin{array}{ccc} T & 0 & 0\\ 0 & T & 0\\ 0 & 0 & T \end{array}\right]\;\left[\begin{array}{c} v_{1}\cos\left(\theta_{a}\right)\\ v_{1}\sin\left(\theta_{a}\right)\\ \left(v_{1}/l\right)\tan\left(v_{2}\right) \end{array}\right]+{\left[\begin{array}{c} x_{a}\\ y_{a}\\ \theta_{a} \end{array}\right]}_{k}$$

This motion model will be used to simulate the motion of the UGV when validating the presented localisation method using numerical simulations. The forward UWB tag and the rear UWB tag are fixed right on the centreline of the UGV, in the front and in the rear of the UGV. Therefore, the middle position of the forward and the rear UWB tags coincides with the exact midpoint of the UGV on the horizontal plane. Hence, the real-world forward UWB tag’s position $\left(x_{f},y_{f}\right)$ and the real-world rear UWB tag’s position $\left(x_{r},y_{r}\right)$ can be found, if $x_{a},y_{a}$ and $\theta_{a}$ are known. Using the solution of (1), $x_{a},y_{a}$ and $\theta_{a}$ can be calculated when simulation parameters $v_{1},\;v_{2},\;l$ and *T* are known. Therefore, simulating the motion of the UWB tags based on the simulated motion of the UGV can be performed.

#### 2.1.2. Motion Model for Localisation and Heading Estimation

For the location and the heading estimation problem, a Continuous Velocity (CV) model [15] can be assumed for a 2D localisation scenario depicted in Figure 1. The CV model has been used by assuming that the localised vehicles are not highly manoeuvred in both linear and angular movements. Let the tag positions’ state vector x be defined as ${\left[x_{f}\;{\dot{x}}_{f}\;y_{f}\;{\dot{y}}_{f}\;x_{r}\;{\dot{x}}_{r}\;y_{r}\;{\dot{y}}_{r}\right]}^{T}$. Hence, when considering the UGV state with respect to the earth-fixed inertial frame, the kinematic equation of the estimation model is given by:

(2) $$\begin{array}{c} {\hat{x}}_{k+1}^{-}=\left[\begin{array}{cccccccc} 1 & T & 0 & 0 & 0 & 0 & 0 & 0\\ 0 & 1 & 0 & 0 & 0 & 0 & 0 & 0\\ 0 & 0 & 1 & T & 0 & 0 & 0 & 0\\ 0 & 0 & 0 & 1 & 0 & 0 & 0 & 0\\ 0 & 0 & 0 & 0 & 1 & T & 0 & 0\\ 0 & 0 & 0 & 0 & 0 & 1 & 0 & 0\\ 0 & 0 & 0 & 0 & 0 & 0 & 1 & T\\ 0 & 0 & 0 & 0 & 0 & 0 & 0 & 1 \end{array}\right]\;{\hat{x}}_{k}^{+}+\nu \\ \begin{array}{ccc} {\hat{x}}_{k+1}^{-} & = & A\;{\hat{x}}_{k}^{+}+\nu \end{array} \end{array}$$

where ν is the process noise. The hat notation depicts that the respective variable is estimated from the localisation algorithm. The subscript *k* denotes the time step, and superscripts “+” and “−” are used to denote the *a posteriori* estimation and the *a priori* estimation, respectively.

Since the observations are taken as the ranges between UWB tags on the UGV and UWB anchors on the drones, the equation of the state dynamics should have to be written relative to the UAVs. Nevertheless, the state dynamics in (2) have not been written relative to the UAVs. By projecting the state of UAVs to the ground plane (as the altitude of the UGV is not used), the following derivation justifies why it is not necessary to use a system dynamics equation written related to UAV frames.

The state of a given UAV, projected on to the ground plane, is defined as ${\;}_{g}u_{n,k};\;n\in \left\{1,2\right\}$, representing either of the UAVs by the subscript *n*. Therefore,

$${\;}_{g}u_{n,k}={\left[\begin{array}{c} x_{n,k}\;{\dot{x}}_{n,k}\;y_{n,k}\;{\dot{y}}_{n,k}\;x_{n,k}\;{\dot{x}}_{n,k}\;y_{n,k}\;{\dot{y}}_{n,k} \end{array}\right]}^{T}$$

If the UGV states are written relative to the ground projected body fixed frames of the UAVs by assuming that the evolution of UAV states can be modelled using a discrete nonlinear/linear transition $b_{n}\left(\;\cdot \;\right)$ of the current state, based on the exact UAV motion model, then Equation (2) can be written as:

(3) $$\begin{array}{ccc} \begin{array}{c} {\hat{x}}_{k+1}^{-} \end{array} & = & \begin{array}{c} A\;{\hat{x}}_{k}^{+}+{\;}_{g}u_{1,k+1}-b_{1}\left({\;}_{g}u_{1,k}\right) \end{array}\\ \begin{array}{c} {\hat{x}}_{k+1}^{-} \end{array} & = & \begin{array}{c} A\;{\hat{x}}_{k}^{+}+{\;}_{g}u_{2,k+1}-b_{2}\left({\;}_{g}u_{2,k}\right) \end{array} \end{array}$$

since the UAV coordinates are known with high accuracy. In ${\;}_{g}u_{n,k}$, it can be seen that the first four elements are identical to the last four elements. This is because, when the UGV states are written relative to the ground projected body-fixed frames of a specific UAV, both front tag elements and rear tag elements in x have to be written relative to that specific UAV.

Based on the assumption that the UAV states’ evolution can be modelled using a discrete nonlinear transition $b_{n}\left(\;\cdot \;\right)$ of the current state based on the exact UAV motion model, the relative state transition of the UGV in (3) reduces to its initial form as in (2). In other words, it is justifiable to use (2) to represent the discrete state transition of the UGV for this range-based localisation, without writing (2) relative to each UAV.

#### 2.1.3. Measurement Model

The measurement model for the EKF is formulated by getting expressions for the squared range measurements using the coordinates of both UAVs and UGVs.

The state of either of the UAVs will be taken as $u_{n,k}={\left[x_{n,k}\;y_{n,k}\;z_{n,k}\right]}^{T}$; $n\in \left\{1,2\right\}$, elements of which will be used in later sections. For simplicity, the time step subscript will be omitted whenever it is insignificant. The observation vector is $h\left(x\right)={\left[h_{1}\;h_{2}\;h_{3}\;h_{4}\right]}^{T}$, where $h_{1},h_{2},h_{3}$ and $h_{4}$ are the squared range measurements.

If $R_{nm}=\sqrt{z_{n}^{2}+{\left(x_{n}-x_{m}\right)}^{2}+{\left(y_{n}-y_{m}\right)}^{2}}$ and $m\in \left\{f,r\right\}$, which is the range between the UGV tag *m* and the UAV anchor *n*, then the overall observation equation can be written as:

(4) $$y=h\left(x\right)=\left[\begin{array}{c} R_{1f}^{2}\\ R_{1r}^{2}\\ R_{2f}^{2}\\ R_{2r}^{2} \end{array}\right]\;+\eta$$

where η is the measurement noise of the range sensors. Since this measurement equation is nonlinear, the Jacobian with respect to the estimation state vector has to be calculated when implementing the EKF using the backward numerical differentiation. Thereupon, the Jacobian of the measurement function h(x), evaluated at *a priori* is given by Equation (5).

(5) $$H{\left.=\;\frac{𝜕h\left(x\right)}{𝜕x}\right|}_{{\hat{x}}_{k}^{-}}$$

### 2.2. Localisation Using the Kalman Filter

Due to the nonlinearity in the observation/measurement model, and also because the localisation is performed sequentially in real-time, the discrete-time version of the EKF [16] is used. In order to use an EKF, the initial ${\hat{x}}_{k-1}^{+}$ and the initial $P_{k-1}^{+}$, which is the error covariance associated with the *a posteriori* estimates ${\hat{x}}_{k-1}^{+}$, has to be provided to the EKF algorithm. A random point near the vicinity of the UGV can be given as the initial ${\hat{x}}_{k-1}^{+}$ for the UGV. Initially, a diagonal matrix, which has fairly high values in the diagonal elements, can be given for $P_{k-1}^{+}$ for all UGVs, indicating the initial value of ${\hat{x}}_{k-1}^{+}$ is substantially uncertain. In the following sections, EKF-related symbols have the usual notation.

Following the estimation from the EKF, the forward UWB tag position estimate $\left({\hat{x}}_{f},{\hat{y}}_{f}\right)$ and the rear UWB tag position estimate $\left({\hat{x}}_{r},{\hat{y}}_{r}\right)$ can be used to find the location (i.e., geometrical centroid) of the UGV and its heading using:

(6) $$\left({\hat{x}}_{a},{\hat{y}}_{a}\right)=\left[\frac{{\hat{x}}_{f}+{\hat{x}}_{r}}{2},\frac{{\hat{y}}_{f}+{\hat{y}}_{r}}{2}\right]$$

(7) $${\hat{\theta }}_{a}=\tan^{-1}\left(\frac{{\hat{y}}_{f}-{\hat{y}}_{r}}{{\hat{x}}_{f}-{\hat{x}}_{r}}\right)$$

as previously mentioned in Section 2.1. In (6), it is assumed that the UWB range sensors are mounted on the UGV centreline from an equal distance from the centre of the UGV.

## 3. Unobservability in Localisation

When observability lapses in a system, the optimal estimators fail in state estimation. If such a situation occurs in localisation, it is defined as an unobservability in localisation. Therefore, a thorough observability analysis is essential in order to guarantee a fail-safe state estimation during the localisation. The next two sections will explain how to identify the localisation singularities/unobservabilities in the UAV–UGV collaborative localisation scenario based on deterministic observability.

### 3.1. Deterministic Observability

The deterministic observability analyses and checks whether the state of a system can be determined without any ambiguity, based on the system’s outputs [17], where a state estimator cannot give an accurate estimate about the system state at a deterministic observability lapsed situation. Nevertheless, the observability of a nonlinear system is not a global attribute in the entire state space as the relationship between the measurement space, and the state space is not one-to-one [18]. Thereupon, deterministic local observability has been defined as: “A system is locally observable at a state $x_{0}$, if there exists a neighbourhood N of $x_{0}$ such that every state, which belongs to N, other than $x_{0}$ is distinguishable from $x_{0}$. Finally, the system is locally observable if it is locally observable at each state” [19].

For a generic affine continuous-time nonlinear system:

(8) $$\begin{array}{cc} \dot{x} & =f\left(x\right)+\sum_{\varrho =1}^{\vartheta }u_{\varrho }g_{\varrho }\left(x\right)\\ y & =h\left(x\right) \end{array}$$

where $x\in R^{\zeta }$ is the system state vector, $y\in R^{\vartheta }$ is the output (observation) vector, $g_{1}\left(x\right),\;g_{2}\left(x\right),\;\cdots \;,\;g_{\vartheta }\left(x\right)$ are known vector fields, and the control input is $u={\left[u_{1}\;\cdots \;u_{\vartheta }\right]}^{T}$; if the current state $\left(x_{0}\right)$ is given and the expression

(9) $$\begin{array}{c} \left(\nabla L_{z_{s}}L_{z_{s-1}}\;\cdots \;L_{z_{1}}\;h_{j}\right)\left(x_{0}\right)\\ s\geq 0\;\;\;\;z_{i}\in \left\{\;f,g_{1},g_{2},...\;,g_{\vartheta }\;\right\} \end{array}$$

is calculated at $x_{0}$, where $L\left(\cdot \right)$ is the Lie derivative, then System (8) is locally observable in the neighbourhood of $x_{0}$ if there are *n* linearly independent row vectors in this set (i.e., full in rank).

Since the proposed localisation method is used for real-time sequential state estimation, achieving deterministic local observability all the time is a requirement to maintain an accurate state estimation without any singularities/ambiguities.

### 3.2. Identification of Singularities

Since our aim is to localise a non-manoeuvring UGV, u in (8) is not known to the estimation algorithm. Henceforth, the expression in (9) reduces to an observability matrix,

(10) $$O\left(x\right)={\left[\frac{𝜕L_{f}^{\;0}h\left(x\right)}{𝜕x}\cdots \;\cdots \;\cdots \;\frac{𝜕L_{f}^{\;\zeta -1}h\left(x\right)}{𝜕x}\right]}^{T}$$

where $L_{f}h\left(x\right)$ is the Lie derivative of the function h(x) by the function f(x) and $L_{f}^{\;\zeta }h\left(x\right)$ is the ${\left(\zeta +1\right)}^{th}$ Lie derivative of the function h(x) by the function f(x).

If the system is locally observable at every time, the rank of O(x) should be equal to *n* (i.e., full rank) [18,19]. In this localisation scenario, O(x) for our system, when written as in (10), is a 32×8 matrix that uses (4) as the observation equation h(x) and the linear continuous form of the difference equation (2) as the system equation f(x) for UGV dynamics. To find the singularities, $O{\left(x\right)}^{T}$ is used for mathematical convenience instead of O(x) using the identity $rank\left(O(x)\right)=rank\left(O{\left(x\right)}^{T}\right)$. By analysing at which UGV states result in loss of full rank of $O{\left(x\right)}^{T}$, the singularities can be identified. The upper triangular matrix of the LU-decomposed O(x) is in the row echelon form [20], therefore if any of its diagonal elements is zero at any system state, O(x) cannot be a full rank matrix, and hence the system becomes locally unobservable.

By analysing the diagonal elements of the upper triangular matrix of the LU-decomposed $O{\left(x\right)}^{T}$, the singularity is identified to be taking place when UGV states satisfy either

(11) $${\left[\begin{array}{c} \left(y_{2}-y_{1}\right)\\ 0\\ \left(x_{1}-x_{2}\right)\\ 0\\ 0\\ 0\\ 0\\ 0 \end{array}\right]}^{T}\cdot {\hat{x}}_{k}=x_{1}y_{2}-x_{2}y_{1}$$

or

(12) $${\left[\begin{array}{c} 0\\ 0\\ 0\\ 0\\ \left(y_{2}-y_{1}\right)\\ 0\\ \left(x_{1}-x_{2}\right)\\ 0 \end{array}\right]}^{T}\cdot {\hat{x}}_{k}=x_{1}y_{2}-x_{2}y_{1}$$

**Figure 2:**
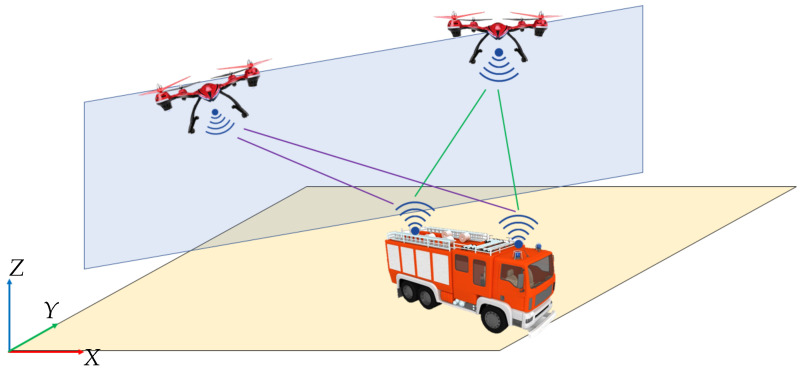
Ambiguity region of the location observer.

Hence, the location where the localisation ambiguity/singularity of the observer occurs according to (11) or (12) is the vertical shaded blue surface in Figure 2, which intercepts both UAV positions.

What happens due to this localisation singularity during a real-time localisation is that when a UGV range sensing tag intercepts the shaded blue surface in Figure 2, the optimal estimator fails to estimate the UGV location, i.e., the optimal estimator cannot determine in which side of the shaded blue surface, the intercepted UGV range sensing tag is at.

## 4. Aversion of Ambiguities

Based on the identified singularities in Section 3, avoiding ambiguity will be of interest in accurately localising the UGVs in real time. Nevertheless, the motion of the UGV and UAVs is subjective to the mission objectives that cannot be altered due to observability issues. Therefore, developing a method which enables a UGV to successfully pass through the ambiguity region in Figure 2 is the key to eradicating the ambiguity errors in localisation. Thereupon, CEKF has been utilised by imposing constraints based on heuristics, covariance-based accuracy margins and inter-dependency of state variables.

Even if the system model depicts the real system to a greater extent, KFs with constraints [21,22,23,24] have been used in estimation problems to improve the estimation accuracy by avoiding the unrealistic state estimates irrespective of the system nonlinearities [21].

In this CEKF-based UAV–UGV collaborative localisation method, the estimation projection method has been used for the state estimation, where the unconstrained *a posteriori* estimate of the EKF ${\hat{x}}_{k}^{+}$ is projected into the constrained space and obtaining the constrained estimate ${\tilde{x}}_{k}^{+}$ by:

(13) $${\tilde{x}}_{k}^{+}=argmin_{x}{\left(x-{\hat{x}}_{k}^{+}\right)}^{T}W\left(x-{\hat{x}}_{k}^{+}\right)$$

such that

Dx=dand/orDx≤d

are the linear equality constraints and linear inequality constraints, respectively.

**Figure 3:**
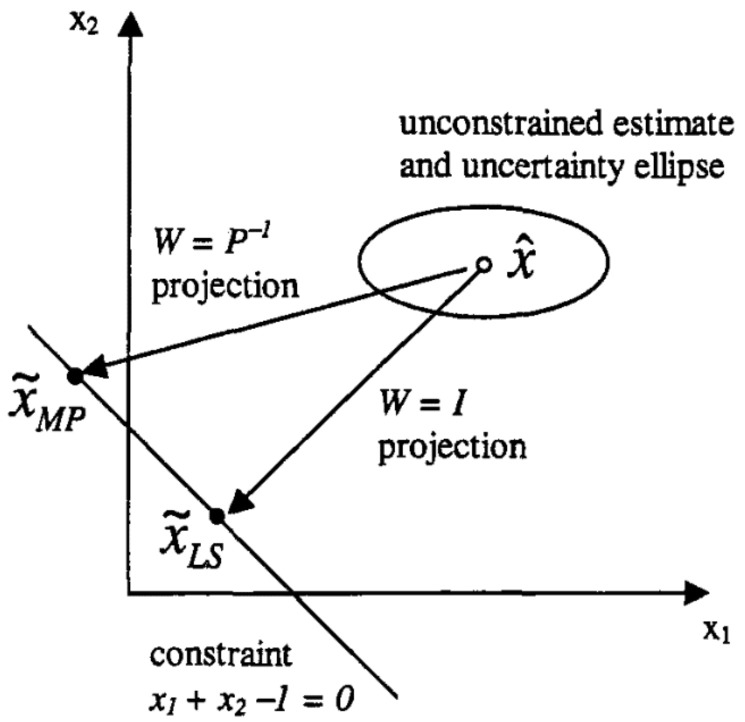
An example which shows the difference between the Maximum Probability (MP) approach and the Least Squares (LS) approach in projecting the estimate into the constraint space [25].

The nonlinear constraints can also be handled in the same way by linearising the nonlinear constraints using the first-order Taylor expansion of the constraints [21,25]. In (13), the matrix W is the weighting matrix. The value of W is equal to the inverse of the *a posteriori* covariance matrix of estimation if the projection is based on the maximum probability approach. If the projection is based on the least-squares approach, the matrix W is equal to the identity matrix. In the proposed localisation method, the maximum probability state projection method is followed ($∴W={\left(P_{k}^{+}\right)}^{-1}$). Due to the maximum probability constraining approach, and also because the EKF is unbiased, the overall CEKF state estimation process is still a minimum variance estimation approach. Figure 3 illustrates the difference between the two projection approaches intuitively.

In Figure 3, the least squares method has been followed to calculate ${\tilde{x}}_{LS}$ by projecting the estimate $\hat{x}$ onto the constraint space. In that method, there is no concern about safeguarding the minimum variance objective of the estimator when imposing constraints.

Imposing constraints to an EKF (i.e., CEKF) is a quadratic optimisation problem as formulated in (13). Active set-based quadratic programming can be utilised to find the constrained estimate ${\tilde{x}}_{k}^{+}$, by identifying the active constraints in each step of the optimisation. Hildreth’s quadratic programming procedure, which is simple and reliable in real-time implementation can be utilised to solve this quadratic optimisation problem [26,27,28].

In the following three sections, the novel constraints, which are the main contributions of this paper, have been formulated with derivations. The symbol $\sigma_{\chi }$ depicts the standard deviation of the a posteriori estimation of a variable χ.

Note: The constraints of the CEKF, explained in this section, are to be imposed only after the EKF’s covariance matrix of estimation is sufficiently converged. All of the constraints are formulated based on ±3σ uncertainty (i.e., 99.73% confidence level).

### 4.1. The Position Constraint

Assuming that the UGV motion can be successfully modelled by the CV motion model, a position constraint can be imposed on the UGV state estimation based on the ±3σ uncertainty of the position caused by the uncertainty of the velocity. For instance, if the estimated position along *x*-direction $x_{f,k-1}$ and the estimated velocity along *x*-direction ${\dot{x}}_{f,k-1}$ of the UGV’s front UWB tag at the previous time step is known, then the position of that tag at this time step can be kinematically anticipated as $x_{f,k-1}+{\dot{x}}_{f,k-1}\;T$. However, $x_{f,k-1}$ and ${\dot{x}}_{f,k-1}$ are stochastic variables. Therefore, deterministic anticipation is inappropriate, and have to take $\sigma_{{\dot{x}}_{f,k-1}}$ to stochastically anticipate the position of that tag. Hence, a compound inequality can be written for that tag as:

(14) $$\begin{array}{c} {\dot{x}}_{f,k-1}\;T\;-\;\;3\sigma_{{\dot{x}}_{f,k-1}}\;T\;\leq \;x_{f,k}\;-\;x_{f,k-1}\leq \;{\dot{x}}_{f,k-1}\;T\;+\;\;3\sigma_{{\dot{x}}_{f,k-1}}\;T \end{array}$$

and a kinematic constraint can be formulated at the $x_{f,k}$ estimation step based on this compound inequality. In such a kinematically constrained EKF-based estimator, $x_{f,k-1}$ and ${\dot{x}}_{f,k-1}$ are constrained estimates, and have to be written as ${\tilde{x}}_{f,k-1}$ and ${\dot{\tilde{x}}}_{f,k-1}$, respectively. Then, $x_{f,k}$ will be the free variable for the optimisation in the CEKF, which was explained in Section 4. Hence, (14) can be modified as:

(15) $${\tilde{\dot{x}}}_{f,k-1}\;T\;-\;\;3\sigma_{{\dot{x}}_{f,k-1}}\;T\;\leq \;x_{f,k}\;-\;{\tilde{x}}_{f,k-1}\leq \;{\tilde{\dot{x}}}_{f,k-1}\;T\;+\;\;3\sigma_{{\dot{x}}_{f,k-1}}\;T$$

The constraints in (15) can be extended to all the other position variables ($x_{r,k},\;y_{f,k}$ and $y_{r,k}$) of the UGV tag positions’ state vector (x) by writing another three compound inequalities analogous to (15). Altogether, all four compound inequalities should be imposed simultaneously in the CEKF, and also, the compound inequalities have to be re-arranged as inequalities such that the consolidated inequality becomes:

(16) $$L\;x\leq M\;{\tilde{x}}_{k-1}+N\;\mathrm{diag}{\left(P_{k-1}^{+}\right)}^{\circ 1/2}$$

where the 8×8 diagonal matrices L,M and N independently have the matrices $L_{0},M_{0}$ and $N_{0}$ as their block diagonal matrices, respectively, where

$$L_{0}=\left[\begin{array}{cc} 1 & 0\\ -1 & 0 \end{array}\right]\;\;,\;M_{0}=\left[\begin{array}{cc} \;1 & \;T\\ -1 & -T \end{array}\right]\;\;,\;N_{0}=\left[\begin{array}{cc} 0 & T\\ 0 & T \end{array}\right],$$

and ${\left(\;\cdot \;\right)}^{\circ \;1/2}$ is the element-wise square root of a matrix.

Further derivations have to be performed to (16) in order to implement its constraints in the form of $D_{1}x\;\leq \;d_{1}$. First, (16), must be re-arranged such that (16) can be obtained as a nonlinear inequality in the form of $g_{i}\left(x\right)\leq b_{i}$, where the $b_{i}$ vector consists of all the variables in (16), which are independent of x. In order to linearise (16), a first-order Taylor expansion at the *a priori* estimate can be used [25]. Hence, the Jacobian of (16) can be written as

(17) $$\begin{array}{cc} g_{i}^{\prime }\left(x\right) & =\frac{𝜕g_{i}\left(x\right)}{𝜕x}\\ & =\left[\begin{array}{cccccccc} 1 & 0 & 0 & 0 & 0 & 0 & 0 & 0\\ -1 & 0 & 0 & 0 & 0 & 0 & 0 & 0\\ 0 & 0 & 1 & 0 & 0 & 0 & 0 & 0\\ 0 & 0 & -1 & 0 & 0 & 0 & 0 & 0\\ 0 & 0 & 0 & 0 & 1 & 0 & 0 & 0\\ 0 & 0 & 0 & 0 & -1 & 0 & 0 & 0\\ 0 & 0 & 0 & 0 & 0 & 0 & 1 & 0\\ 0 & 0 & 0 & 0 & 0 & 0 & -1 & 0 \end{array}\right] \end{array}$$

Using the techniques presented in [21,24,25], (16) can be written as a linear inequality as

(18) $$g_{i}^{\prime }\left(\;{\hat{\;\;x}}_{k}^{-}\right)\;x\;\leq \;b_{i}-g_{i}\left(\;{\hat{\;\;x}}_{k}^{-}\;\right)\;+g_{i}^{\prime }\left(\;{\hat{\;\;x}}_{k}^{-}\;\right){\hat{\;\;x}}_{k}^{-}$$

in the form of $D_{1}x\;\leq \;d_{1}$.

### 4.2. The Heading Constraint

**Figure 4:**
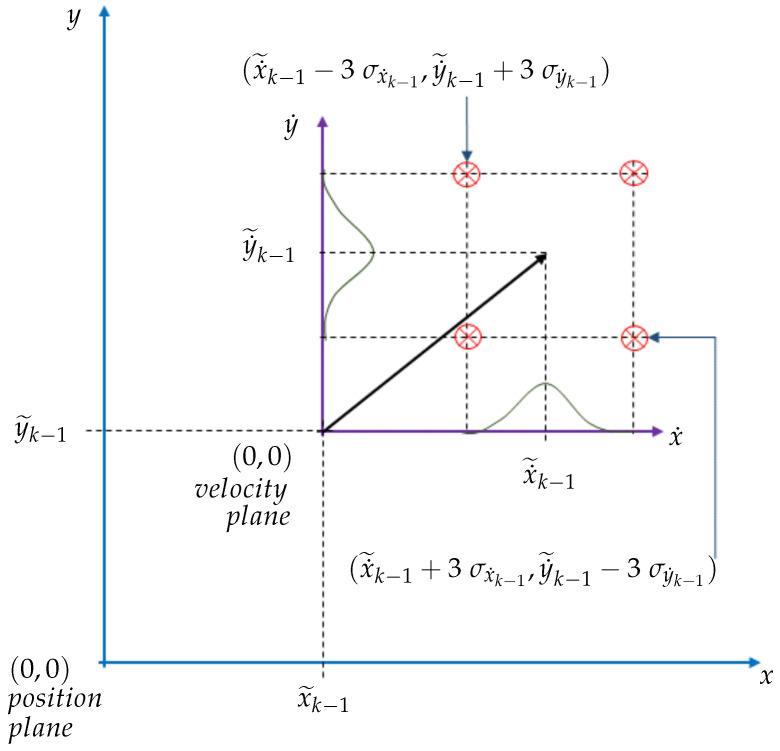
Illustration of the heading constraint.

Assuming that the UGV motion can be modelled by the CV motion model, a heading constraint can be imposed based on the 3σ uncertainty of the heading of a UGV range sensing tag, based on a UGV tag’s velocity triangle. This is a constraint which is based on the kinematics of the UGV on a planar terrain. For simplicity, first, the subsequent derivation was performed by considering a given UWB tag on the UGV as a particle moving on a Cartesian plane. In Figure 4, the position plane is the Cartesian coordinate frame, relative to which a UGV tag’s positions are denoted. Moreover, the velocity plane is the Cartesian coordinate frame, relative to which a UGV tag’s velocity vectors are denoted. The velocity plane is an instantaneous coordinate frame, the origin of which is placed on the respective UGV tag’s estimated position $\left({\tilde{x}}_{k-1},\;{\tilde{y}}_{k-1}\right)$ at the previous time step. The velocity of a UGV tag is drawn in a solid black arrow, as shown in Figure 4 on the velocity plane.

(19) $$\begin{array}{c} \tan^{-1}\left(\frac{{\tilde{\dot{y}}}_{k-1}-\;3\sigma_{{\dot{y}}_{k-1}}}{{\tilde{\dot{x}}}_{k-1}+\;3\sigma_{{\dot{x}}_{k-1}}}\right)\leq \tan^{-1}\left(\frac{y_{k}-{\tilde{y}}_{k-1}}{x_{k}-{\tilde{x}}_{k-1}}\right)\leq \tan^{-1}\left(\frac{{\tilde{\dot{y}}}_{k-1}+\;3\sigma_{{\dot{y}}_{k-1}}}{{\tilde{\dot{x}}}_{k-1}-\;3\sigma_{{\dot{x}}_{k-1}}}\right) \end{array}$$

(20) $$\begin{array}{c} \frac{{\tilde{\dot{y}}}_{k-1}-\;3\sigma_{{\dot{y}}_{k-1}}}{{\tilde{\dot{x}}}_{k-1}+\;3\sigma_{{\dot{x}}_{k-1}}}\leq \frac{(y_{k}-{\tilde{y}}_{k-1})/T}{(x_{k}-{\tilde{x}}_{k-1})/T}\leq \frac{{\tilde{\dot{y}}}_{k-1}+\;3\sigma_{{\dot{y}}_{k-1}}}{{\tilde{\dot{x}}}_{k-1}-\;3\sigma_{{\dot{x}}_{k-1}}}\\ \frac{{\tilde{\dot{y}}}_{k-1}-3\;\sigma_{{\dot{y}}_{k-1}}}{{\tilde{\dot{x}}}_{k-1}+3\;\sigma_{{\dot{x}}_{k-1}}}\leq \frac{{\dot{y}}_{k}}{{\dot{x}}_{k}}\leq \frac{{\tilde{\dot{y}}}_{k-1}+3\;\sigma_{{\dot{y}}_{k-1}}}{{\tilde{\dot{x}}}_{k-1}-3\;\sigma_{{\dot{x}}_{k-1}}} \end{array}$$

When $x,\;y,\;\dot{x}$ and $\dot{y}$ denote the *x*-position of a tag, *y*-position of a tag, the velocity of a tag along the *x*-axis and the velocity of a tag along the *y*-axis, respectively; if an assumption is made (for simplicity) that the direction of a UGV tag’s velocity is in the first quadrant of the velocity plane as shown in Figure 4, then the compound inequality (19) can be obtained. In the compound inequality (19), the expression

$$\tan^{-1}\left(\frac{y_{k}-{\tilde{y}}_{k-1}}{x_{k}-{\tilde{x}}_{k-1}}\right)$$

denotes the direction angle of the relative position vector of the current tag position estimate with respect to the previous tag position estimate. Nevertheless, this direction angle cannot be a deterministic variable due to the uncertainty of the velocity estimates. Referring to the velocity plane in Figure 4, the maximum and the minimum angle of the estimated velocity vector at the previous time step can be easily identified based on the velocity estimation uncertainties’ 3σ edge limits. Hence, the left-hand side expression and the right-hand side expression of the compound inequality (19) can be formulated. Furthermore, (19) can be simplified to (20), where a compound inequality can be obtained, which can be used to impose as a constraint on the UGV state’s velocity variables in the EKF-based localisation.

If the compound inequality constraint in (20) is generalised such that the constraint can be imposed while the UGV tag’s velocity is in any quadrant of the velocity plane, then a common pattern can be observed. Hence, a consolidated inequality expression for the front UGV tag can be written as

(21) $$\begin{array}{ccc} W_{f} & \left[\begin{array}{c} x_{f_{k}}\\ {\dot{x}}_{{\;}_{f_{k}}}\\ y_{f_{k}}\\ {\dot{y}}_{f_{k}} \end{array}\right] & <\left[\begin{array}{c} 0\\ 0 \end{array}\right] \end{array}$$

where

$$W_{f}\;=\;\left[\begin{array}{cccc} 0 & w_{f_{12}} & 0 & w_{f_{14}}\\ 0 & w_{f_{22}} & 0 & w_{f_{24}} \end{array}\right]$$

and the elements of $W_{f}$ are:

$$\begin{array}{c} w_{f_{12}}\;=-\;\left(\;{\tilde{\dot{y}}}_{f,k-1}+\;3\sigma_{{\dot{y}}_{f,k-1}}\;\mathrm{sgn}\left({\tilde{\dot{x}}}_{f,k-1}\right)\right)\\ w_{f_{22}}\;=\;\;\;\;\left(\;{\tilde{\dot{y}}}_{f,k-1}-\;3\sigma_{{\dot{y}}_{f,k-1}}\;\mathrm{sgn}\left({\tilde{\dot{x}}}_{f,k-1}\right)\right)\\ w_{f_{14}}\;=\;\;\;\;\left(\;{\tilde{\dot{x}}}_{f,k-1}-\;3\sigma_{{\dot{x}}_{f,k-1}}\;\mathrm{sgn}\left({\tilde{\dot{y}}}_{f,k-1}\right)\right)\\ w_{f_{24}}\;=-\;\left(\;{\tilde{\dot{x}}}_{f,k-1}+\;3\sigma_{{\dot{x}}_{f,k-1}}\;\mathrm{sgn}\left({\tilde{\dot{y}}}_{f,k-1}\right)\right)\; \end{array}$$

If (21) is extended to both tags of the UGV, a linear inequality in the form of $D_{2}\;x\leq d_{2}$ can be obtained, which can be written as

(22) $$\left[\begin{array}{cc} W_{f} & 0\\ 0 & W_{r} \end{array}\right]x<\left[\begin{array}{c} 0\\ 0\\ 0\\ 0 \end{array}\right]$$

where $W_{r}$ is the equivalent weight matrix in (21) written for the rear tag of the UGV.

### 4.3. Node Separation Constraint

Since the UGV is a rigid body and the localisation sensor nodes are rigidly attached to the UGV in a specific separation distance, a heuristic equality constraint can be imposed based on this aspect. If the localisation tag positions’ coordinates are taken from the UGV state, the equality constraint can be formulated as

(23) $${\left(\;x_{f}-\;x_{r}\right)}^{2}\;+{\;(\;y_{f}-y_{r}\;)}^{2}\;=\;l^{2}$$

in the form of $g_{e}\left(\;x\;\right)=b_{e}$, where l is the sensor node separation distance. In this research, it is assumed that the vehicle length is equal to the sensor node separation. In order to linearise (23), first-order Taylor expansion at the *a priori* estimate can be used, such that

$$g_{e}^{\prime }\left(\;x\;\right)=\frac{𝜕g_{e}}{𝜕x}={\left[\begin{array}{c} 2\left(x_{f}-x_{r}\right)\\ 0\\ 2\left(y_{f}-y_{r}\right)\\ 0\\ -2\left(x_{f}-x_{r}\right)\\ 0\\ -2\left(y_{f}-y_{r}\right)\\ 0 \end{array}\right]}^{T}$$

Hence, the linearised equality constraint can be obtained in the form of $D_{e}\;x\;=\;d_{e}$ as follows:

$$g_{e}^{\prime }\left(\;{\hat{\;\;x}}_{k}^{-}\;\right)\;x\;=\;b_{e}-g_{e}\left(\;{\hat{\;\;x}}_{k}^{-}\;\right)\;+g_{e}^{\prime }\left(\;{\hat{\;\;x}}_{k}^{-}\;\right)\;{\hat{\;\;x}}_{k}^{-}$$

In order to successfully localise the UGV without undergoing any localisation singularities/ambiguities, the three constraints which are discussed have to be imposed concurrently in the CEKF-based UAV–UGV collaborative localisation, where the final expression encompassing all the constraints will be:

(24) $$\begin{array}{cc} \left[\begin{array}{c} D_{1}\\ D_{2} \end{array}\right]x & \leq \left[\begin{array}{c} d_{1}\\ d_{2} \end{array}\right]\\ D_{e}x & =d_{e} \end{array}$$

## 5. Observability Enhancement of the CEKF

Tolerance to the singularities should be an essential feature in the proposed CEKF, in order to maintain the accuracy of localisation. Therefore, observability improvement has to be analysed while the proposed constraints in the previous subsection are imposed in an EKF-based localisation. Apart from the deterministic observability, which is used to analyse fully deterministic systems (or by assuming a system is fully deterministic), stochastic observability is used to analyse the reliability in CEKF for localisation in this particular system as the system is deterministically unobservable.

Stochastic observability: different to a deterministic system, a system can be observable, theoretically at least, if an appropriate random process is driving the system [29]. The random process can also be influenced by a reverse effect [29].

Stochastic observability implies that there exists a state estimator/filter of which the state estimation variance is bounded [30]. Hence, we can accept the state estimation of the CEKF if the error covariance bound of the proposed CEKF is sufficient in eradicating the state ambiguity in estimation.

Stochastic observability of the CEKF is analysed in real-time using the posterior Cramér–Rao bound. The most common version of the posterior Cramér–Rao bound in the context of Kalman filters is the bound, which is computed using the Fisher information matrix. However, calculating the estimation error covariance bound using the Fisher information matrix is incorrect as the state estimation is constrained in the CEKF. Therefore, the posterior Cramér–Rao bound must be computed in real-time, and then compensation must be made to the result to reflect the effects of the CEKF constraints on the estimation error covariance bound.

### 5.1. Posterior Cramér–Rao Bound

The information of a given estimator is defined as the inverse of the covariance matrix of estimation [31]. Hence, the information matrix $J_{k}$ of a given estimator is defined as the inverse of the estimation error covariance matrix [32]:

(25) $$J_{k}={\left(P_{k}\right)}^{-1}$$

On the other hand, PCRB has been defined as

(26) $$PCRB=E\left\{\left[g\left(\bar{y}\right)-\bar{x}\right]{\left[g\left(\bar{y}\right)-\bar{x}\right]}^{T}\right\}\geq J_{k}^{-1}$$

where $g(\bar{y})$ is a function of the observation vector $\bar{y}$, which delivers the output of a state estimator. The output vector of the function $g(\bar{y})$ is an estimate of the state vector $\bar{x}$. From (25) and (26), it can be seen that the covariance matrix of estimation that can be obtained using an estimation algorithm is bounded by the PCRB. Moreover, for an unbiased estimator such as the Kalman filter, Equation (26) becomes an equality [31].

In a real-time estimation process, calculating the PCRB directly using the expected values in (26) cannot be performed, because $\bar{x}$ is not available. Therefore, PCRB should be calculated using the probability distribution functions of the state and the estimation of the state instead. If $p_{\bar{y},\bar{x}}\left(\bar{y},\bar{x}\right)$ is the joint probability density of $\bar{y}$ and $\bar{x}$, then the elements of the information matrix at a given time step are such that [32]:

(27) $$J_{ij}=E\;\left[-\;\frac{{𝜕}^{2}\;\;\log\;p_{\bar{y},\bar{x}}\left(\bar{y},\bar{x}\right)}{𝜕{\bar{x}}_{i}𝜕{\bar{x}}_{j}}\right]\;\;i,\;j=1,\;\cdots \;,n$$

provided that the expectations and derivatives exist.

To calculate PCRB at time step *k*, if we define state vector ${\bar{x}}_{k}=\left[x_{1},\cdots ,x_{k}\right]$ and the observation vector ${\bar{y}}_{k}=\left[y_{1},\cdots ,y_{k}\right]$, where $x_{1},\cdots ,x_{k}$ and $y_{1},\cdots ,y_{k}$ are a sequence of states and a sequence of measurements/observations of a non-linear state estimation process, respectively; then, the joint probability distribution $p(\bar{x},\bar{y})$ in (27) can be expressed as:

(28) $$p_{{\bar{x}}_{k},{\bar{y}}_{k}}\left({\bar{x}}_{k},{\bar{y}}_{k}\right)=p\left(x_{0}\right)\prod_{j=1}^{k}p\left(y_{j}|x_{j}\right)\prod_{i=1}^{k}p\left(x_{i}|x_{i-1}\right)$$

From (28), it is apparent that the $p_{{\bar{x}}_{k},{\bar{y}}_{k}}\left({\bar{x}}_{k},{\bar{y}}_{k}\right)$ expression is expanding as the time increases. Hence, if $p_{{\bar{x}}_{k},{\bar{y}}_{k}}\left({\bar{x}}_{k},{\bar{y}}_{k}\right)$ is used directly to calculate J, the computational cost will increase as the time passes [31,32]. If there is a recursive calculation method to calculate the PCRB at the $k^{th}$ time step, using the PCRB of the time step k−1, it will be a more computationally efficient way than the aforementioned way of calculating the PCRB.

In [31], a Riccati-like information matrix calculation method has been proposed, which is a great achievement since the method helps the sequential calculation of the PCRB efficiently with lower computational power in real-time estimation processes. Based on the recursive PCRB calculation methods presented in [31], the information matrix J can be calculated for a nonlinear time-invariant system with additive Gaussian noises (υ and η), such as:

(29) $$\begin{array}{c} x_{k+1}=f_{k}\left(x_{k}\right)+\upsilon \\ y_{k}=h_{k}\left(x_{k}\right)+\eta \end{array}$$

using

(30) $$\begin{array}{cc} J_{k+1} & ={\left(Q_{k}+A_{k}J_{k}^{-1}A_{k}^{T}\right)}^{-1}+H_{k+1}^{T}R_{k+1}^{-1}H_{k+1}\\ \mathrm{where}\\ A & =\frac{𝜕f(\cdot )}{𝜕x}\\ H & =\frac{𝜕h(\cdot )}{𝜕x} \end{array}$$

when A and B are evaluated at the *a posteriori* estimate.

### 5.2. Constrained Posterior Cramér–Rao Bound

The constrained EKF method, which is used in this research (estimation projection), which has been explained in Section 4, is a minimum variance and unbiased method [22,33]. In the CEKF, the *a posteriori* state estimate is projected into the state space, which is feasible with respect to the constraints. Hence, the information addition during both the a posteriori estimation and constrained estimation have to be evaluated at the CEKF state estimation update. In [34], it has been shown that the classical PCRB is invalid for the constrained state estimation and, therefore, a modified PCRB has been derived for constrained state estimations. Moreover, it has been shown in [34,35] that only the active constraints are contributing to the decrement of the constrained PCRB. The constraints are functions of state variables and system parameters. If an estimator has *c* number of active equality constraints at a given time step, such that

(31) $$G_{c}=\left[\begin{array}{c} g_{1}\left(\cdot \right)\\ g_{2}\left(\cdot \right)\\ \vdots \\ g_{c}\left(\cdot \right) \end{array}\right]=0$$

where only the active constraints are denoted by $g_{1}\left(\cdot \right),g_{2}\left(\cdot \right),\cdots ,g_{c}\left(\cdot \right)$, then the CPCRB for any unbiased constrained state estimator is defined as

(32) $$CPCRB\;=C_{c}\;J^{-1}$$

where

(33) $$C_{c}=I-J^{-1}{\left[\nabla G_{c}\right]}^{T}{\left\{\left[\nabla G_{c}\right]J^{-1}{\left[\nabla G_{c}\right]}^{T}\right\}}^{+}\left[\nabla G_{c}\right]$$

Note: In (33), ${\left\{\cdot \right\}}^{+}$ is the pseudo-inverse of a matrix and the gradient ∇ has to be calculated with respect to the state vector. In this research, the constraints $G_{1},G_{2}$ and $G_{3}$ are taken as the sensor node separation-based constraint, position constraint and heading constraint, respectively, whenever they become active constraints.

### 5.3. Observability Analysis of the Proposed CEKF-Based Localisation

**Figure 5:**
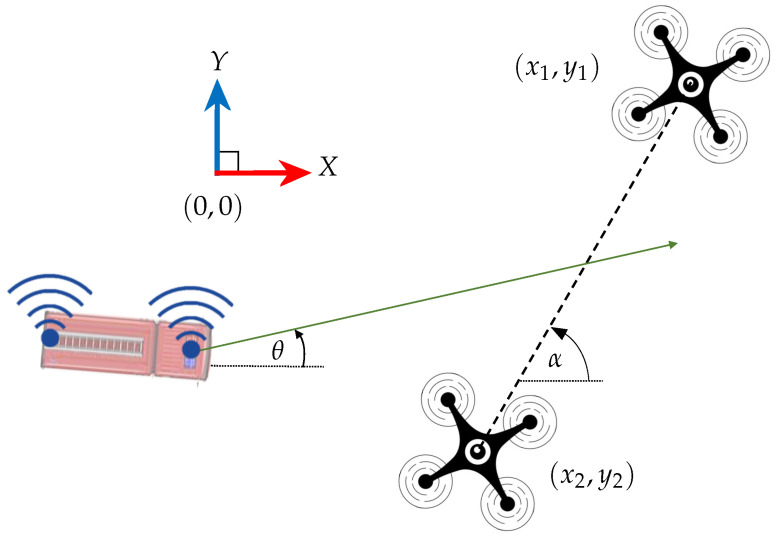
Plan view of the UAV–UGV system.

Using the CPCRB, the variance of a given UWB tag’s estimated travel direction θ can be calculated so that reliable navigation can be ensured if

(34) $$3\sigma_{\theta }\;<\;\left(\alpha -\theta \right)$$

at every time step since the CEKF itself restricts the state estimation to $\pm 3\sigma_{\theta }$ as explained in Section 4 (see Figure 5). A safer operation can be ensured by incorporating a safety factor $k_{sf}>1$ into this condition, such that Equation (34) will become:

$$3k_{sf}\sigma_{\theta }\;<\;\left(\alpha -\theta \right)$$

by providing the CEKF more robustness against uncertainties.

From (34), it is apparent that calculating $\sigma_{\theta }$ is the first step to assess the ambiguity aversion capability of the CEKF. For this purpose, we adopt the following relationship:

(35) $$\sigma^{2}\left(\tan\left(\theta \right)\right)=\sigma^{2}\left(\frac{{\tilde{\dot{y}}}^{+}}{{\tilde{\dot{x}}}^{+}}\right)$$

where $\sigma^{2}\left(\cdot \right)$ is the variance.

**Figure 6:**
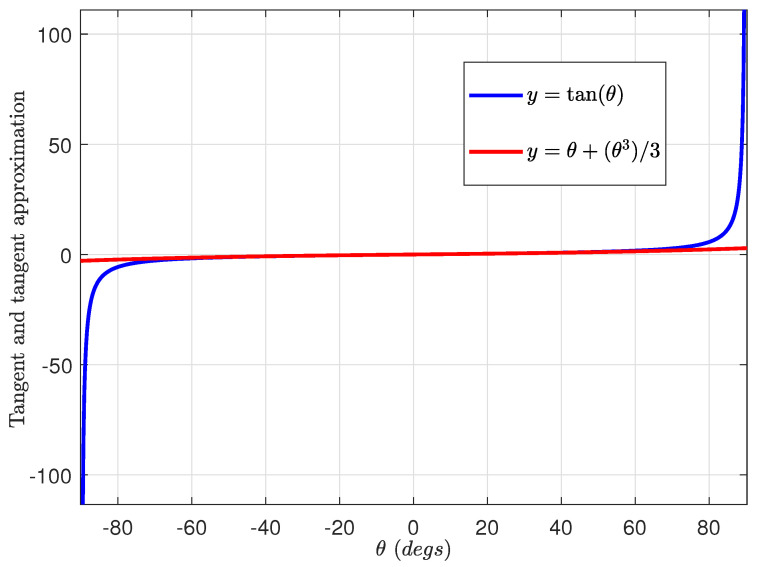
tan(θ) approximation function.

Using the series expansion for $\tan\left(\theta \right)$ when $\left|\theta \right|\leq 45{}^{\circ}$, (35) can be approximated by

(36) $$\sigma^{2}\left(\theta +\frac{\theta^{3}}{3}\right)\approx \sigma^{2}\left(\frac{{\tilde{\dot{y}}}^{+}}{{\tilde{\dot{x}}}^{+}}\right)$$

where the tan(·) approximation function behaves as shown in Figure 6.

Assuming the heading angle estimation’s probability distribution is a Gaussian distribution, the LHS of Equation (36) can be estimated using the moment generating functions (non-central). The mean value of θ (i.e., $\mu_{\theta }$), which has to be known to be able to follow this method, can be calculated by

$$\mu_{\theta }=\tan^{-1}\left(\frac{{\tilde{\dot{y}}}^{+}}{{\tilde{\dot{x}}}^{+}}\right)$$

using the constrained *a posteriori* estimates. The RHS of (36) can be calculated using the Taylor series approximated variance of ratios [36]:

(37) $$\sigma^{2}\left(\frac{{\tilde{\dot{y}}}^{+}}{{\tilde{\dot{x}}}^{+}}\right)={\left(\frac{{\tilde{\dot{y}}}^{+}}{{\tilde{\dot{x}}}^{+}}\right)}^{2}\left[\frac{\sigma^{2}\left({\tilde{\dot{y}}}^{+}\right)}{{\left({\tilde{\dot{y}}}^{+}\right)}^{2}}+\frac{\sigma^{2}\left({\tilde{\dot{x}}}^{+}\right)}{{\left({\tilde{\dot{x}}}^{+}\right)}^{2}}-2\frac{\mathrm{cov}\left({\tilde{\dot{y}}}^{+},{\tilde{\dot{x}}}^{+}\right)}{{\tilde{\dot{x}}}^{+}\cdot {\tilde{\dot{y}}}^{+}}\right]$$

The value of $\sigma^{2}\left({\tilde{\dot{y}}}^{+}/{\tilde{\dot{x}}}^{+}\right)$ in (37) can be calculated using the constrained estimations of the CEKF.

After calculating the LHS of (36) using moment-generating functions, (36) can be written as:

(38) $$\frac{5}{3}\sigma^{6}\left(\theta \right)+\left(2+4\mu_{\theta }^{2}\right)\sigma^{4}\left(\theta \right)+(1+\mu_{\theta }^{2}{)}^{2}\;\sigma^{2}\left(\theta \right)\approx \sigma^{2}\left(\frac{{\tilde{\dot{y}}}^{+}}{{\tilde{\dot{x}}}^{+}}\right)$$

Hence, the solution to Equation (36) is a cubic equation, roots of which are $\sigma_{\theta }^{2}$.

Solving Equation (38) as a cubic polynomial equation, $\sigma_{\theta }^{2}$ can be calculated by only taking the positive real roots for granted. The value of $\sigma_{\theta }$ can be used to prove the efficacy of this CEKF-based localisation methodology, as mentioned earlier in this section. Moreover, $\sigma_{\theta }$ can be used as a safety indicator to alarm the UAVs to adjust the α angle by moving appropriately if $\alpha -\theta \leq 3\sigma_{\theta }$, which can lead to an erroneous localisation.

Caveat: Since the approximation in Equation (36) is not valid when $45{}^{\circ}<\left|\theta \right|\leq 90{}^{\circ}$, an intermediate coordinate transformation must be used in order to solve Equations (35)–(38), which can be reversed after obtaining the equations’ solutions without affecting the CEKF/EKF algorithms.

## 6. Simulation Results

The numerical simulations were performed to localise a UGV from UAVs using the CEKF method. Furthermore, the ambiguity aversion efficacy is tested for the simulated scenarios. In the simulations and experiments, the algorithmic workflow explained in Algorithm 1 was executed:
**Algorithm 1** CEKF-based UAV–UGV collaborative localisation algorithminitialise the EKF;**do**     obtain range measurements;     estimate the UGV location using the EKF;**while** *EKF estimation covariance matrix is not converged***loop**     obtain range measurements     estimate the UGV location using the EKF;     calculate the constrained location estimation;     calculate $\sigma_{\theta }$;     **if** $\left(\alpha -\theta \right)\leq 3k_{sf}\sigma_{\theta }$ **then**           move the UAVs to adjust α;     **end****end loop**

The simulation results are presented in the next two subsections.

### 6.1. Ambiguity Aversion by CEKF Method

Numerical simulations were carried out to assess a UGV localisation scenario on a two-dimensional plane using two stationary UAVs. The UGV is supposed to drive through the unobservable boundary in Figure 2.

**Figure 7:**
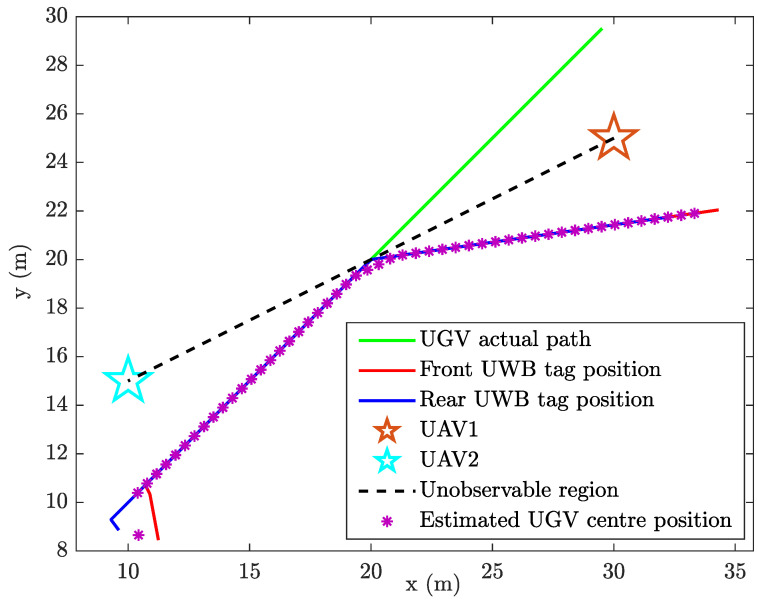
Unconstrained localisation of the UGV.

Unconstrained EKF shows an error in localisation at the unobservable boundary (Figure 7), which ultimately results in a substantial localisation error, i.e., the mirror image of the actually traversed trajectory is given as the EKF estimated location. This happens because the range readings are identical for the real trajectory and its mirror image trajectory.

**Figure 8:**
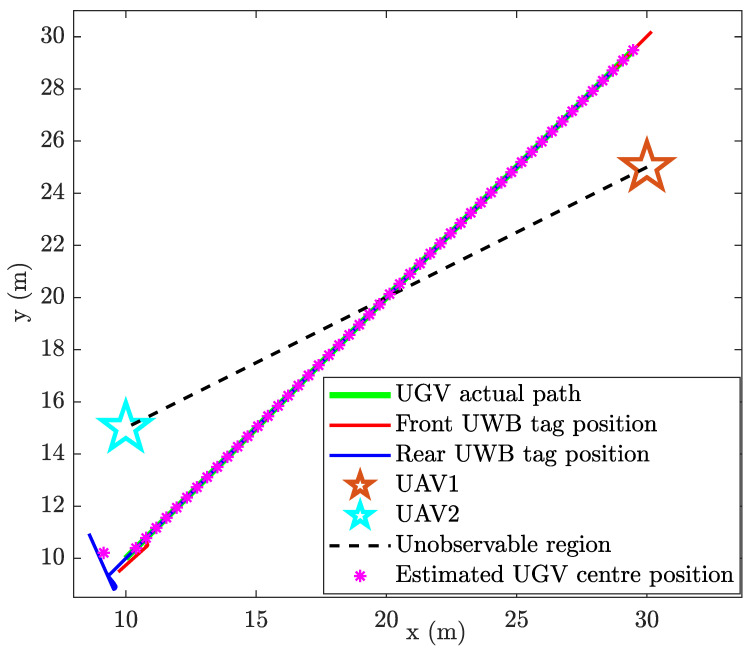
Constrained localisation of the UGV.

**Figure 9:**
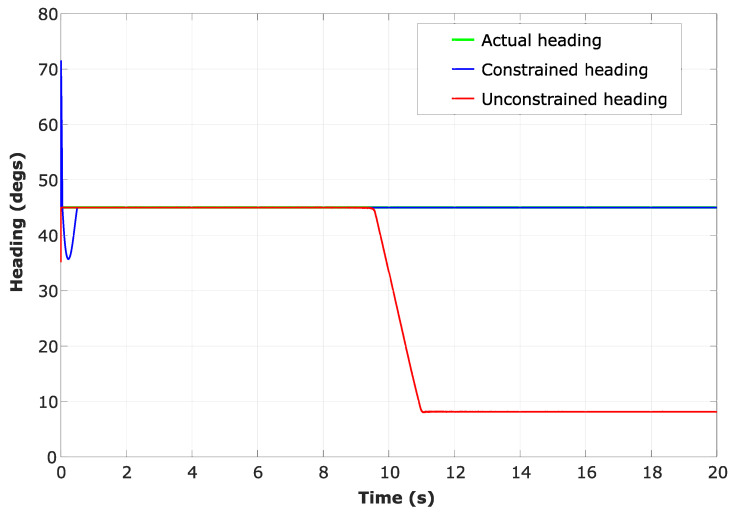
Estimated heading of a tag, which is fixed on the UGV.

In an identical situation, the state-constrained localisation from CEKF does not show an error in localisation at the line of unobservability (Figure 8). Figure 9 shows the heading angle (θ) estimation result, which affirms that there have not been any singularities occurred during the CEKF-based localisation.

Another numerical simulation was performed where the UGV travels along a lengthy two-dimensional random path requiring the UAVs to move, i.e., the UAVs are also moving while the UGV localisation is carried out. CEKF is used to estimate the location of the UGV. In this simulation, the UGV successfully traverses across the line of unobservability twice without any erroneous localisation due to singularities.

**Figure 10:**
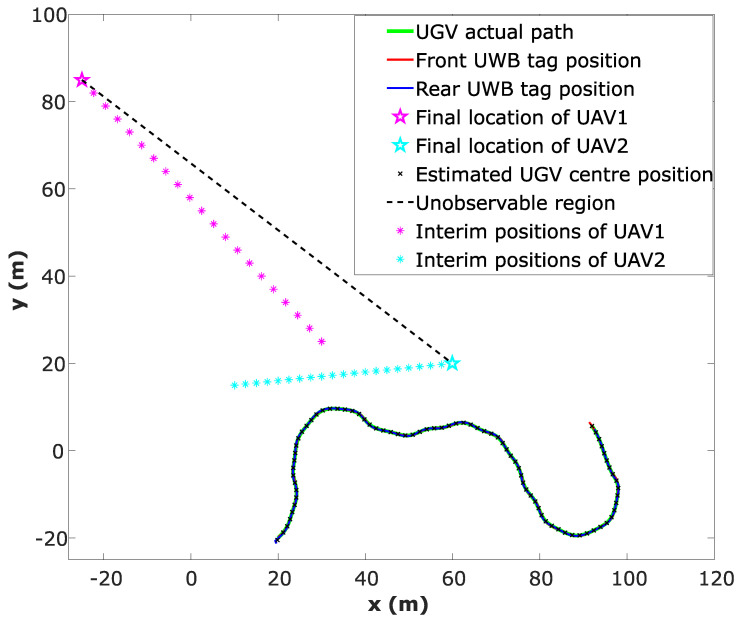
Constrained localisation of the UGV along a random path using the CEKF-based localisation method.

**Figure 11:**
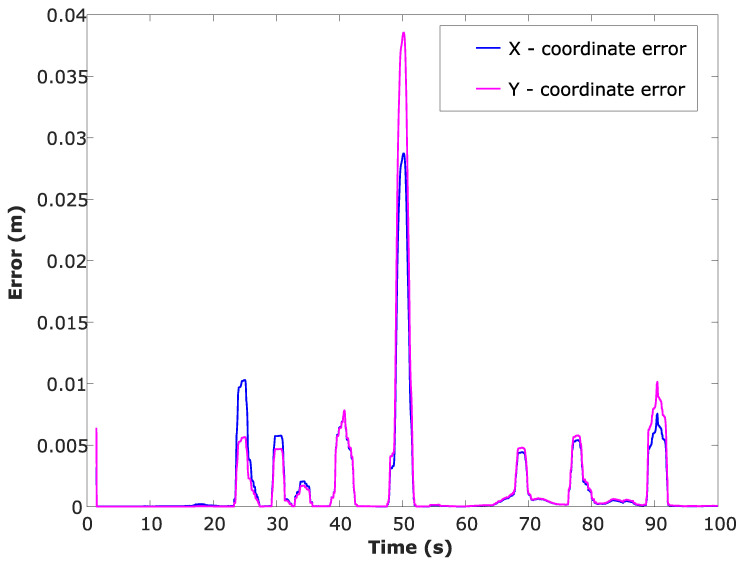
RMSE along the random travel path when the UGV is localised using the CEKF-based localisation method.

Location estimation plot of the UGV, ground truth locations of the UGV and the respective UAV positions are shown in Figure 10. Root Mean Squared Error (RMSE) has been calculated while the UGV is travelling along the random path and the RMSE vs. time for both *x* and *y* coordinates is shown in Figure 11.

**Figure 12:**
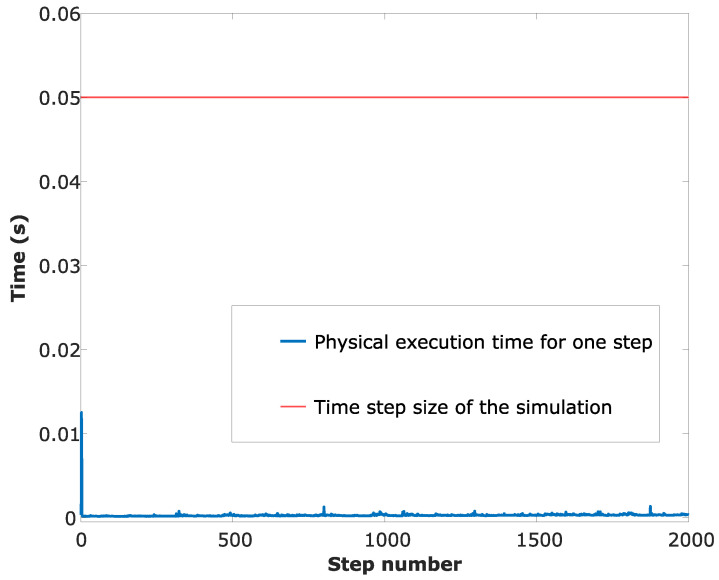
Real-time performance of the CEKF localisation algorithm.

The sampling time of the simulation is 50 ms, which is typically an attainable step time in field vehicles’ (e.g., unmanned fire truck) onboard computers while other peripheral devices are also operated/controlled by the same onboard computer. In order to measure the real-time performance of the CEKF localisation algorithm, the code was run in MATLAB2018b software and the physical time was measured for each time-step of the simulation using the inbuilt stopwatch timer facility (MATLAB commands: *tic, toc*). At the beginning of each iteration of the simulation, the *tic* command is called to record the physical time, and at the end of each iteration, the *toc* command is called to record the physical time. Using the time difference of the recorded physical timings, the iteration execution time is calculated. Based on performance results, which are plotted in Figure 12, it can be seen that the physical real-time calculations can be performed without any time lags to successfully localise a UGV using the presented CEKF-based localisation algorithm.

### 6.2. Observability of CEKF

**Figure 13:**
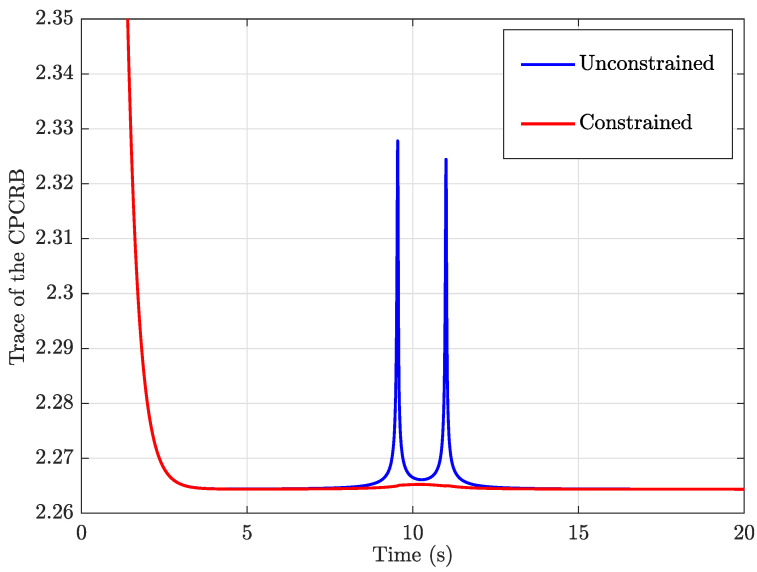
Trace of the CPCRB in unconstrained EKF and constrained EKF.

The diagonal of the CPCRB consists of the lower bounds of state estimation error variances for each and every element in the estimated state vector. Hence, the trace of the CPCRB matrix is an indicator of stochastic observability, i.e., smaller the trace value, the higher the stochastic observability and vice versa. The trace of the CPCRB calculated for the EKF localisation and for the CEKF localisation shows a difference in the trace of the covariance matrix of estimation error lower bound (i.e., CPCRB), as shown in Figure 13. Two peaks shown in blue show the increment of stochastic unobservability when the two UWB tags intercept the unobservable blue-coloured vertical plane shown in Figure 2. In contrast, the CEKF method has successfully minimised the adverse effects of unobservability.

**Figure 14:**
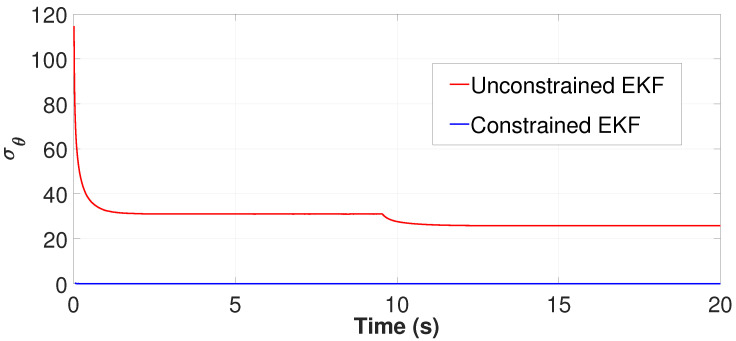
Estimated $\sigma_{\theta }$, which is derived from the CPCRB.

In this simulation, α was kept constantly at 26.6° by keeping the UAVs still, and θ was also kept constant at 45° by driving the UGV with a constant heading for simplicity. Figure 9 and Figure 14 show a more steady θ estimated value despite the unobservability when the CEKF method is used for the localisation. However, when the unconstrained EKF is used for the localisation, a drastic deviation of the heading estimation is notable at 9.5s when we observe the red curve of Figure 9. It happens at the same time when the UGV undergoes unobservability, as we can see that the first unobservability peak in Figure 13 has also taken place at 9.5 s. Moreover, when CEKF is used, $\sigma_{\theta }$ is significantly low without any sudden changes, even at/after the instance when the unobservability occurs. According to Figure 14, $\sigma_{\theta }$ is 31.1° before encountering the unobservability, and 23.9° after encountering the unobservability when using the unconstrained EKF. On the other hand, $\sigma_{\theta }$ is steadily maintained around 0.05°; when using the CEKF. Since $\sigma_{\theta }$ is around 0.05°, the condition for ambiguity aversion: $\alpha -\theta >3\sigma_{\theta }$ (in (34)) is also strongly satisfied in this simulation. Hence, we can conclude that the CEKF has mitigated the ambiguities in localisation and contributed to increasing the localisation accuracy.

## 7. Experiment Results

**Figure 15:**
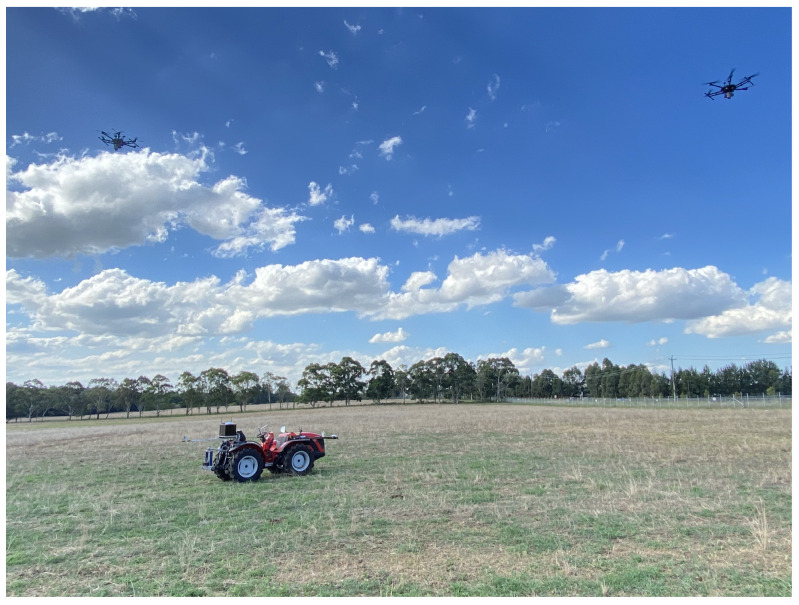
UAV–UGV range-based collaborative localisation experiment.

An experiment was performed to validate the CEKF-based UAV–UGV collaborative localisation method. Two DJI-M600Pro hexacopters (UAVs) were used for range measurement, position estimation and heading estimation of a stationary Antonio Carraro farm tractor (UGV). In Figure 15, an image of the overall experiment is shown. The experiment was performed on a farmland in Menangle, NSW, Australia.

**Figure 16:**
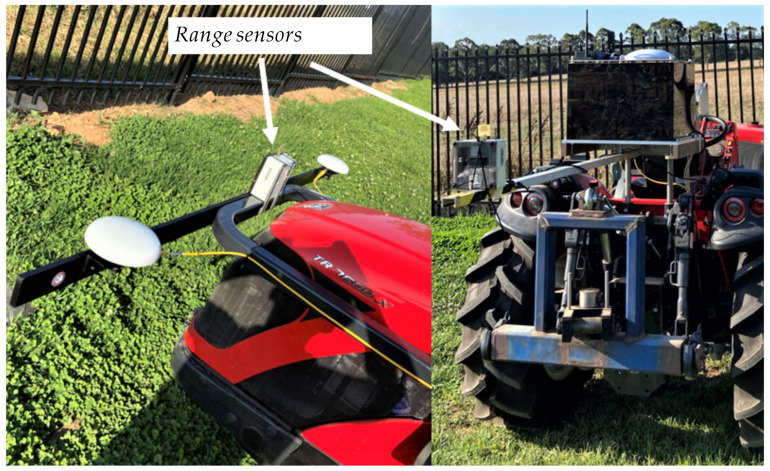
Range sensors on the UGV.

**Figure 17:**
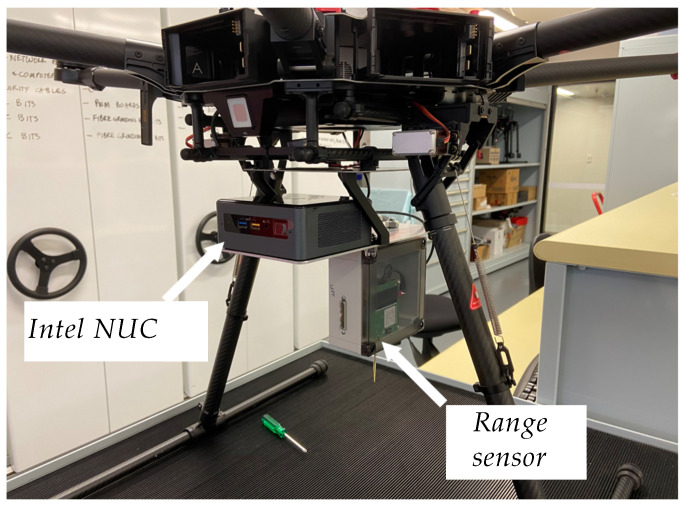
Range sensors on the UAV.

The TREK-1000 range sensors manufactured by Decawave, Ireland were mounted on the front side of the vehicle and on the rear side of the UGV, as shown in Figure 16. The range sensors were mounted underneath the UAVs, as shown in Figure 17. Data logging and processing in each UAV were performed in Intel NUC onboard computers, which were fixed on each UAV as shown in Figure 17.

The next two sub-sections will discuss the results of a static UGV localisation experiment and a dynamic UGV localisation experiment.

### 7.1. Stationary UGV Localisation

**Figure 18:**
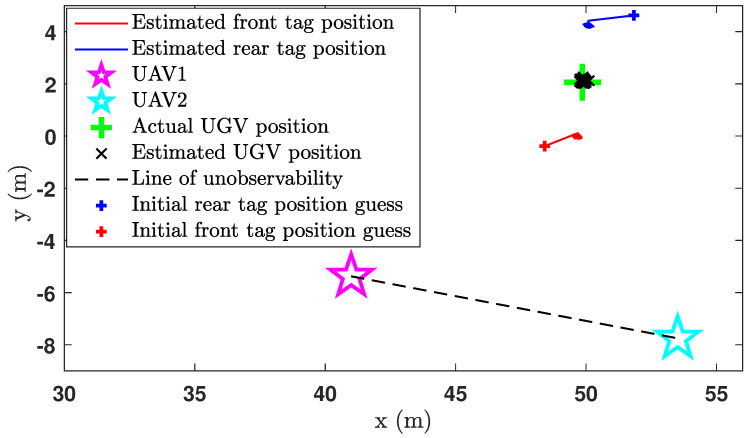
Position estimation plot of the CEKF-based UAV–UGV collaborative localisation when the UGV is not moving.

While the UGV was stationary on the ground, the two UAVs were airborne, and the localisation began while the vehicles were maintaining their positions. While the localisation is in progress, the ground truth location and the heading of the UGV were recorded by the onboard computer of the UGV using an RTK GPS unit. The accuracy of the RTK GPS unit was ±2cm. Figure 18 shows the estimated positions of the UGV during the experiment.

**Figure 19:**
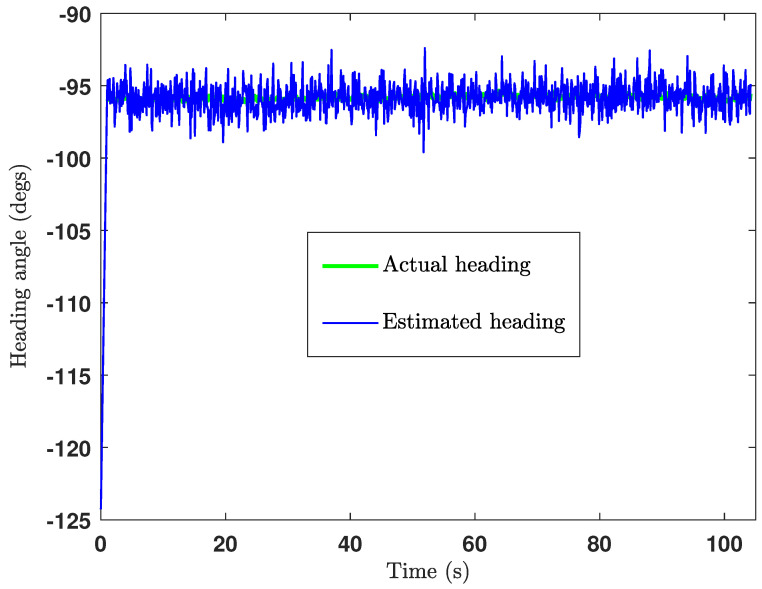
Heading estimation plot of the CEKF-based UAV–UGV collaborative localisation.

**Figure 20:**
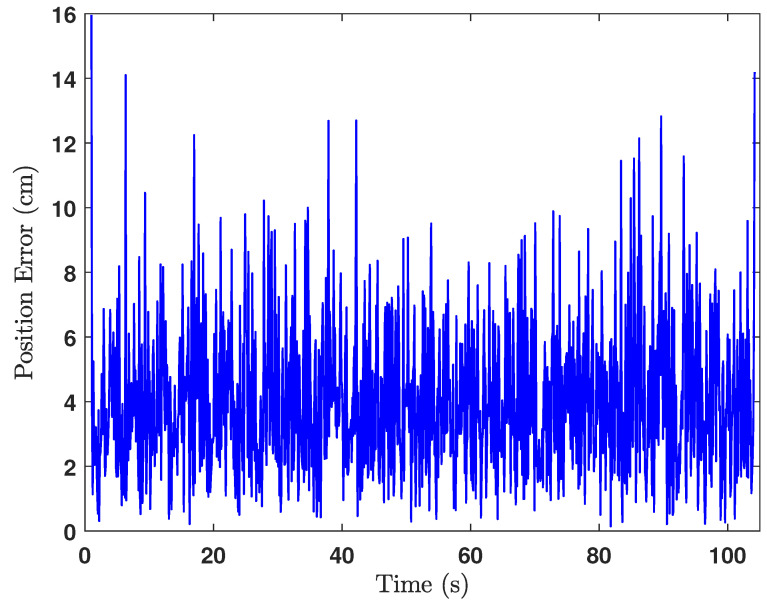
Error of the position estimation.

The green cross in Figure 18 is the ground truth middle position of the UGV, where the estimated middle position of the UGV is shown by the black cross marks. Actually, the middle position of the UGV was calculated at each iteration after estimating the positions of the front UWB tag and the rear UWB tag. The heading of the UGV was also estimated in the same way as shown in Figure 19, from which the heading estimation accuracy can be deduced as ±4°. The UGV positioning error during the experiment is shown in Figure 20, which shows the positioning error of the localisation is less than 14cm.

### 7.2. Non-Stationary UGV Localisation

**Figure 21:**
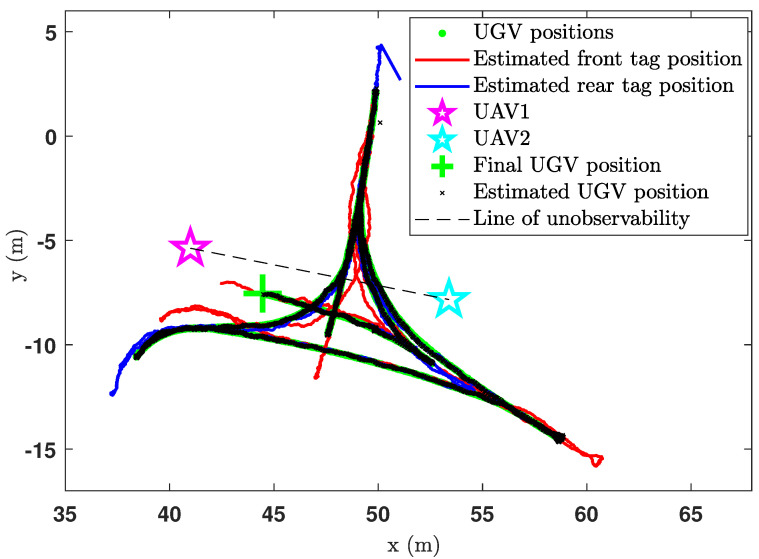
Position estimation plot of the CEKF-based UAV–UGV collaborative localisation when the UGV is moving.

This is a continuation of the experiment discussed in the previous section; thus same experimental instruments have been used. Initially, the UGV was stationary for 105s, while the UAVs are airborne. Thereafter, the UGV started moving along the path marked by green dots in Figure 21. During the UGV movements, the UGV intercepted the line of unobservability several times, during which the CEKF-based localisation algorithm was able to successfully deal with the unobservability problem that was described earlier in Section 3. The CEKF-based localisation algorithm has localised the UGV from its starting position (49.88,2.11) until its final location, which is marked by the green cross in Figure 21.

At the interceptions of the unobservable boundary, the CEKF constraints that are activated prevent the localisation algorithm from producing unrealistic location estimations which are against the kinematics of the UGV. For example, if the location estimation algorithm delivers a false location estimation due to the unobservability (as shown in Figure 7), where the false location estimation is on the mirror image path of the actual trajectory, then the constrains will identify it as an erroneous result and deliver the kinematically feasible correct location estimation. Hence, there have not been any erroneous localisation taken place during field experiments. Nevertheless, if the unconstrained EKF is used, erroneous location estimations will not be rectified as such.

**Figure 22:**
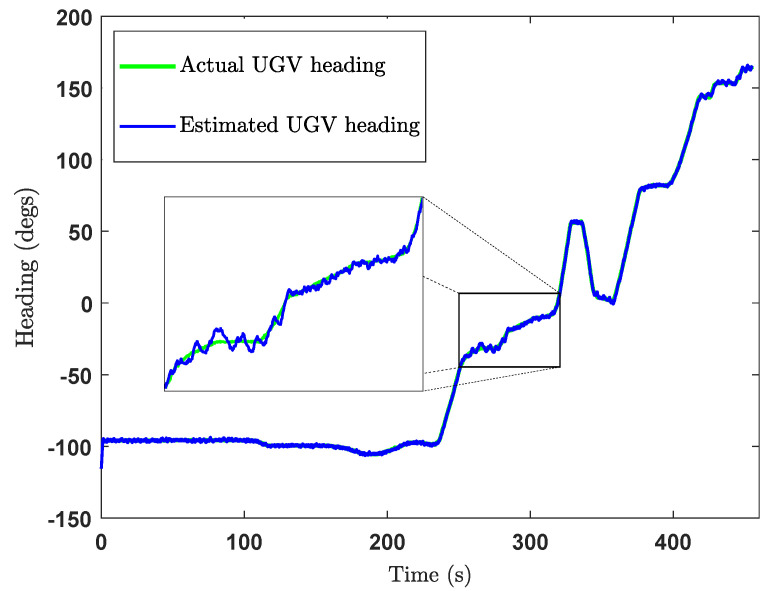
Heading estimation plot of the CEKF-based UAV–UGV collaborative localisation when the UGV is moving.

**Figure 23:**
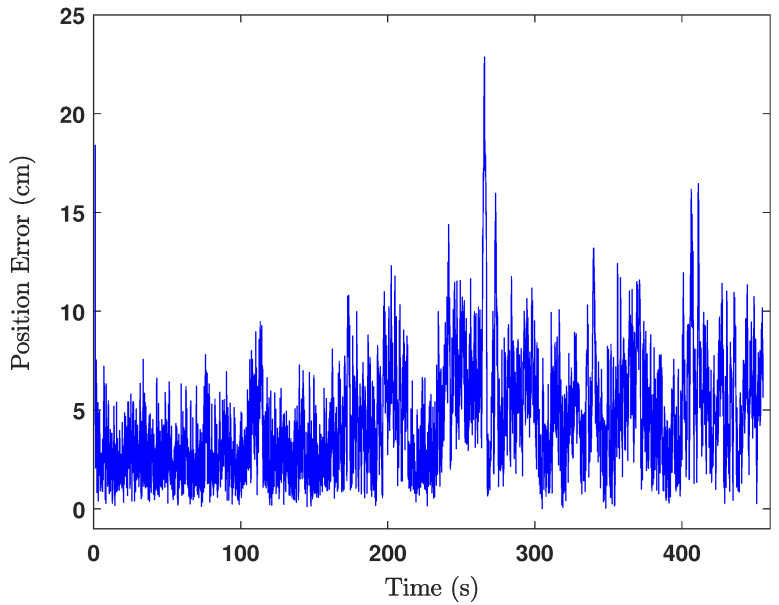
Error of the position estimation when the UGV is moving.

The estimated UGV headings and the positions are as depicted in Figure 22 and Figure 23, respectively. By observing Figure 22 and Figure 23, we can deduce that the heading estimation accuracy is ±4° and the position estimation accuracy is ±23cm.

## 8. Conclusions and Future Works

In a UAV–UGV collaborative localisation with range-only observations, severe localisation errors can occur due to the unavailability of UAVs to be deployed to make sufficient range observations. It is well known that a unique global localisation to track a vehicle on a 2D terrain is possible when three or more drones are deployed to make range-only observations. However, when only two UAVs are deployed, there exists an unobservable region within which it is difficult to localise the UGVs using range-only observations when EKF is used.

The presented CEKF-based localisation method successfully avoids the ambiguity in localisation due to the unobservability. Successful numerical simulations and field experiments show the efficacy in localisation despite the localisation unobservability/ambiguity arising when only two UAVs are deployed for the collaborative localisation task.

The presented CEKF-based localisation method successfully localise a UGV travelling on a 2D terrain, using only two UAVs if an initial position guess (fairly close enough to the UGV) is provided. Nevertheless, the initial position guess must be in the same side where the actual UGV is at; with respect to the unobservability boundary (in Figure 2). After this initial position guess is provided, the subsequent location estimations will converge to the UGV location. Localising a UGV travelling on a 2D terrain, using less than three UAVs cannot be done due to the estimation singularities/ambiguities which may occur during localisation. While the location estimation is ongoing, the ambiguity aversion features of the presented localisation algorithm will rectify any localisation errors whenever the UGV encounters a localisation ambiguity. Because of that, localising a UGV travelling on a 2D terrain, using less than three UAVs is possible with the CEKF-based localisation method presented.

The stochastic observability analysis, which has been presented in this paper, gives solid evidence of how the CEKF-based UAV–UGV collaborative localisation method can overcome estimation singularities. CEKF-based localisation increases the stochastic observability of the localisation more than the EKF-based localisation method. Furthermore, the kinematic constraints, which take the state variables’ variances, give an assurance of successful localisation despite the localisation singularity almost all the time.

However, there can be quite a few instances where UAVs must be slightly re-positioned to ensure a successful localisation. The CPCRB-based stochastic observability analysis framework, discussed in Section 5.3 can be used to identify the instances when the UAVs must be slightly re-positioned.

In conclusion, a UGV which is travelling on a horizontal plane can be localised (and the heading can also be estimated) by only two UAVs using the presented CEKF-based range-only localisation method.

In future works, the proposed system can be further researched to analyse localisation efficacy (1) for different range measurement sensitivities, (2) when occasional range sensing occlusions occur, and (3) in localising UGVs travelling in hilly terrains.

## Data Availability

Data are contained within the article.
